# Supplementary material for: Aesthetics and neural network image representations
Source: Sci Rep. 2023 Jul 15;13:11428. doi: 10.1038/s41598-023-38443-9 (PMC10349859; doi:10.1038/s41598-023-38443-9)
Supplement: Supplementary file 1 — Supplementary Information. [file 41598_2023_38443_MOESM1_ESM.pdf]

A Supplementary information

A.1 Genericity

The figures S1 and S2 shown on the following pages are analogues of Fig. 3 and Fig.5 but obtained for neural networks constructed using a *consecutive* range of random seeds 0-23 used for generating either the multiplicative random perturbations or the weights in block $\mathscr{B}_2$. Since there was no hand-picking of the particular random deformations, in this way one can assess the genericity of the results discussed in the main text. In order to fit all the 24 neural networks, only two types of images are shown: a *stupa* and an *espresso*.

A.2 Dependence on the deformation parameter

The values of the deformation parameter α appearing in the multiplicative deformation given by Eq. (3) in the main text can be linked to a measure of distance in the 55-million dimensional space of BigGAN neural network parameters. A standard measure in such a high dimensional setting is the cosine distance defined by

$$cosine\ distance(x,y) = 1 - cosine\ similarity(x,y) \equiv 1 - \frac{x \cdot y}{|x||y|} \tag{A1}$$

where the vectors in the present context are the concatenated flattened weight tensors of the neural networks. In Fig. S4(left) we show a relation between the α parameter and the cosine distance of the deformed network from the original BigGAN network (for a given choice of random tensors in Eq. (3)). Due to high dimensionality, this relation is essentially identical for other choices of random perturbations. Since in such a high dimensional setting, the cosine distance between any random vectors is very close to 1, we are dealing here with a local neighbourhood of the original network.

In Fig. S3, we show for a range of examples the dependence of the generated images as a function of the deformation parameter α, while keeping the random tensors in Eq. (3) fixed. In the range $\alpha = 0.25 - 0.4$, the departure from the original photo-realistic depictions is already quite noticeable, while still retaining some relation to the original objects.

In order to quantify the above subjective observation, we evaluated the perceptual similarity of variants of a given image differing in the value of α to the four human-made artworks from the ArtBench dataset shown in Fig. 4 in the main text. The results are presented in Fig. S4(right). The perceptual similarity is measured using a pretrained ResNet-152 network (see *Methods*). The generated images used for the comparison are variants of the image in Fig. 4a(left), some of them shown in the top row of Fig. S3.

We observe an increase in similarity around the range of α mentioned above. It is interesting to note, that this increase occurs not only for the artwork identified as the closest to this image (Fig. 4a(right)), but is also quite noticeable for two of the three other artworks. This provides a quantitative argument supporting the observation made in the present paper of the appearance of an *"artistic rendition"*.

A.3 The `StyleGAN-XL` neural network

We repeated the experiment with the multiplicative deformations for a different generative neural network trained on ImageNet — the `StyleGAN-XL` network. We took exactly the same value of the deformation parameter $\alpha = 0.35$ and generated images of a *stupa*, *espresso*, *dial telephone* and *seashore* for deformations with random seeds in the range 0-10. The images were generated with the same value of the truncation parameter 0.4 as default for `BigGAN` (see *Methods*) – for the two networks the truncation parameters are not directly comparable but serve a similar role decreasing the variability of the image latents so as to obtain less pathologies in the images generated by the original networks.

Images generated by a selection of 5 networks (with seeds in the range 0-10), together with the images generated by the original undeformed `StyleGAN-XL` network are shown in Fig. S5, which is a counterpart of Fig. 3 in the main text. We observe that the generated images also appear closer to an *"artistic rendition"* of the original images, even though the deformation parameter was not modified for the new neural network architecture. An interesting feature of these images is that they are somewhat less varied and less "impressionistic" than the ones generated by the `BigGAN` network discussed in the main text. This indicates an imprint of the overall generative neural network architecture, which must be reflected in the way that the visual properties of the photo-realistic world are encoded in the neural network parameters.

A.4 Selected statistical properties of the generated images

It is instructive to compare some statistical properties of the images generated by the deformed networks with the ones produced by the original undeformed `BigGAN` network as well as with real images of the corresponding objects or scenes. As data for the comparison we take generated images for 50 random neural network deformations (of both variants given by Eq. (3) and

Eq. (4) in the main text), 50 images of each kind generated by the original network, and 50 images of each kind from the validation set of the ImageNet dataset (the latter are centrally cropped to a square format and resized to 256×256 resolution).

We examine the distribution of the mean magnitude of Fourier coefficients as a function of the wave-number $|k|$. It is a classical result that this distribution follows a power-law for natural images. In Fig. S6(left), we show the ratio

$$\frac{\langle |Fourier(k)| \rangle}{\langle |Fourier_{natural}(k)| \rangle} \tag{A2}$$

of the mean magnitude to the reference value evaluated for natural images. The generated images have RGB values essentially in the whole range 0-255, so they are not artificially darker or lighter than the natural ones.

We observe that the two types of deformations significantly differ in the lowest wave-numbers. The multiplicative deformations have less pronounced large-scale structures than the natural ones, while the opposite holds for the scrambling of block $\mathscr{B}_2$. This is in agreement with the intuitive identification of the multiplicative case with the *"artistic rendition"* character, and the latter ones being more akin to surrealistic imagery. Note that the undeformed network follows closely the natural images in this range of k. Another interesting feature is that the multiplicative spectra falls off slower than the other cases at intermediate wave numbers.

Another perspective on the loss of detail seen in images generated by the multiplicative deformations is the loss of sharp edges. In order to quantify this, we define *"edginess"* by evaluating a Canny edge detector (as implemented in the `scikit-image` library with $\sigma = 3$ for detecting more significant edges) and taking the mean of the output:

$$edginess \equiv mean(Canny(grayscale(image))) \tag{A3}$$

The results are shown in Fig. S6(right). In accordance with the intuitive expectations, we see an overall decrease for the images generated by networks with multiplicative deformations.

A.5 Nonuniform multiplicative deformations

The multiplicative deformation of the network given by Eq. (3) has a common deformation parameter α. Of course, it is possible to vary this parameter as a function of the network layer. A particularly interesting case would be to enhance the deformation in block $\mathscr{B}_2$, since it upsets the semantic meaning of the various objects while preserving some relatively sharp local features (see Fig. 5 in the main text and Fig. S2 here).

In Fig. S7, we compare the uniform multiplicative deformation with $\alpha = 0.35$ as defined by Eq. (3) with two variations. In the middle column we show deformations acting only on $\mathscr{B}_2$ with $\alpha = 2$, while in the right column in addition we turn on deformations with $\alpha = 0.35$ for all remaining layers.

We observe that the additional $\alpha = 0.35$ deformation softens and smoothens the depictions in the middle column as could indeed be expected. We see, however, that the painterly character is reduced to some degree. This may occur due to the fact that the downstream processing by the mildly deformed layers following $\mathscr{B}_2$ does not act on encodings of natural images but rather on the strongly deformed encodings coming out of $\mathscr{B}_2$, which significantly mixes the natural ingredients.

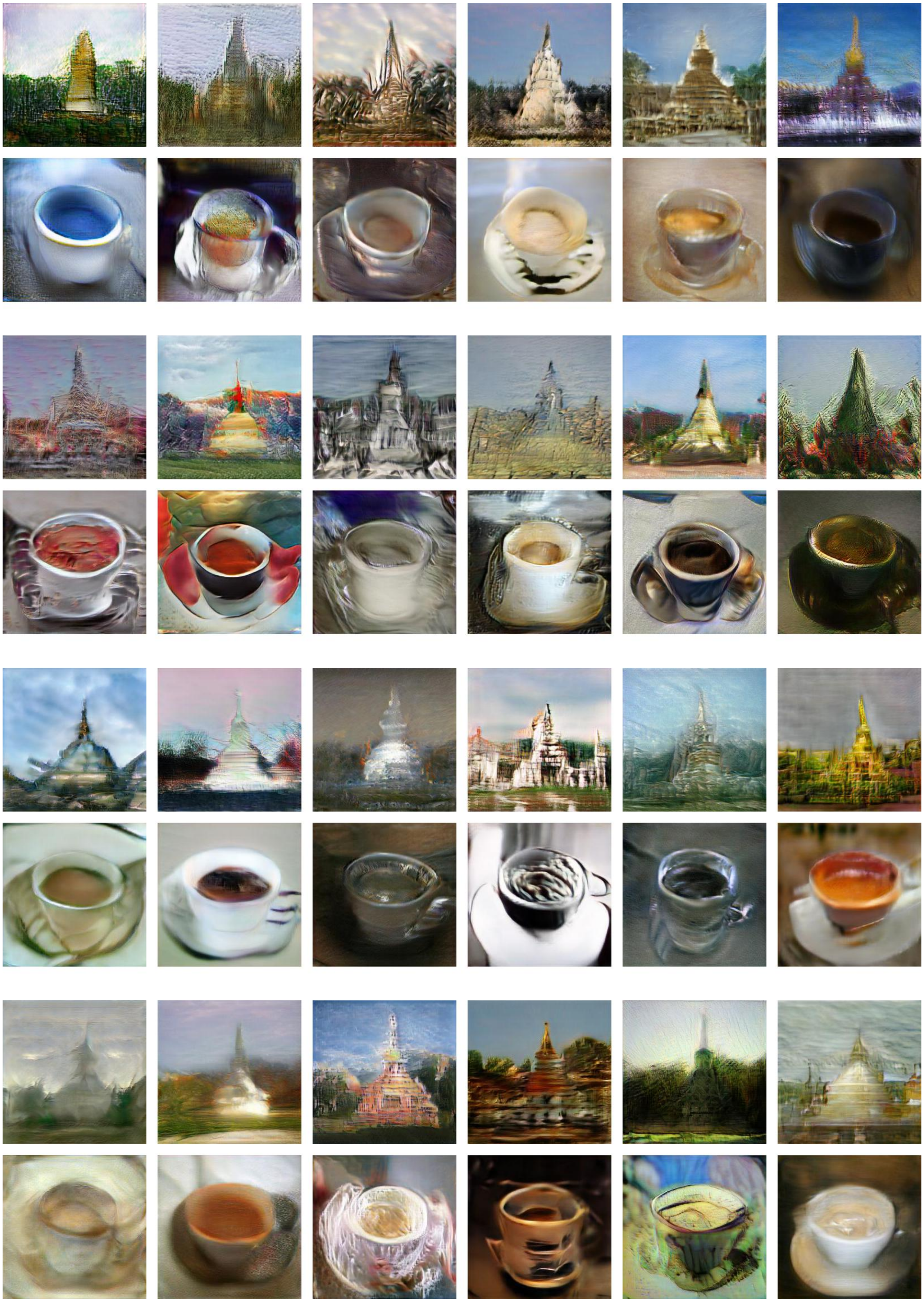

Figure S1. Images of a *stupa* and an *espresso* generated by neural networks with weights obtained through random multiplicative perturbations (Eq. (3) in the main text) for consecutive random seeds 0-23.

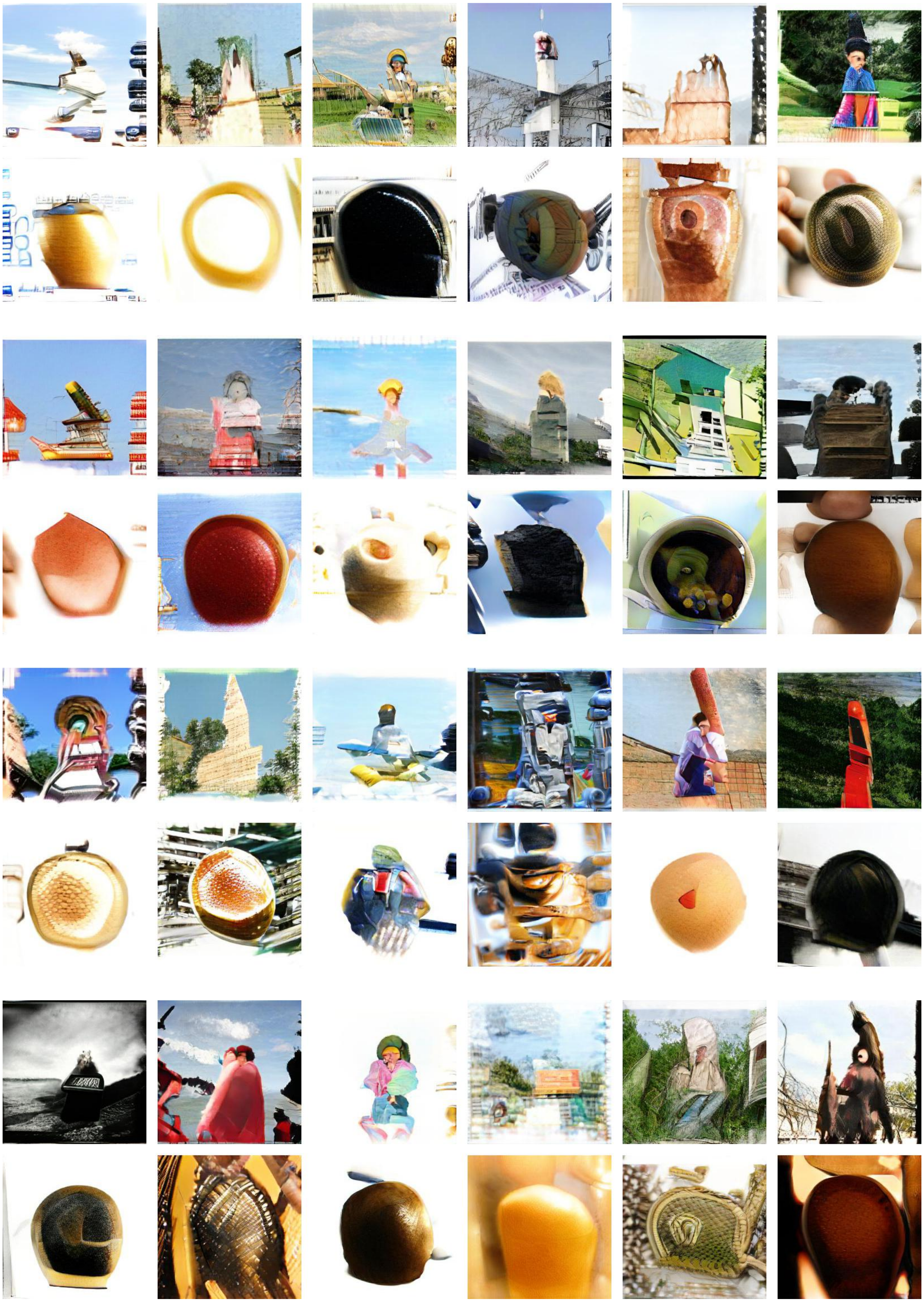

Figure S2. Images of a *stupa* and an *espresso* generated by neural networks with weights obtained through randomly scrambling the block $\mathscr{B}_2$ (Eq. (4) in the main text) for consecutive random seeds 0-23.

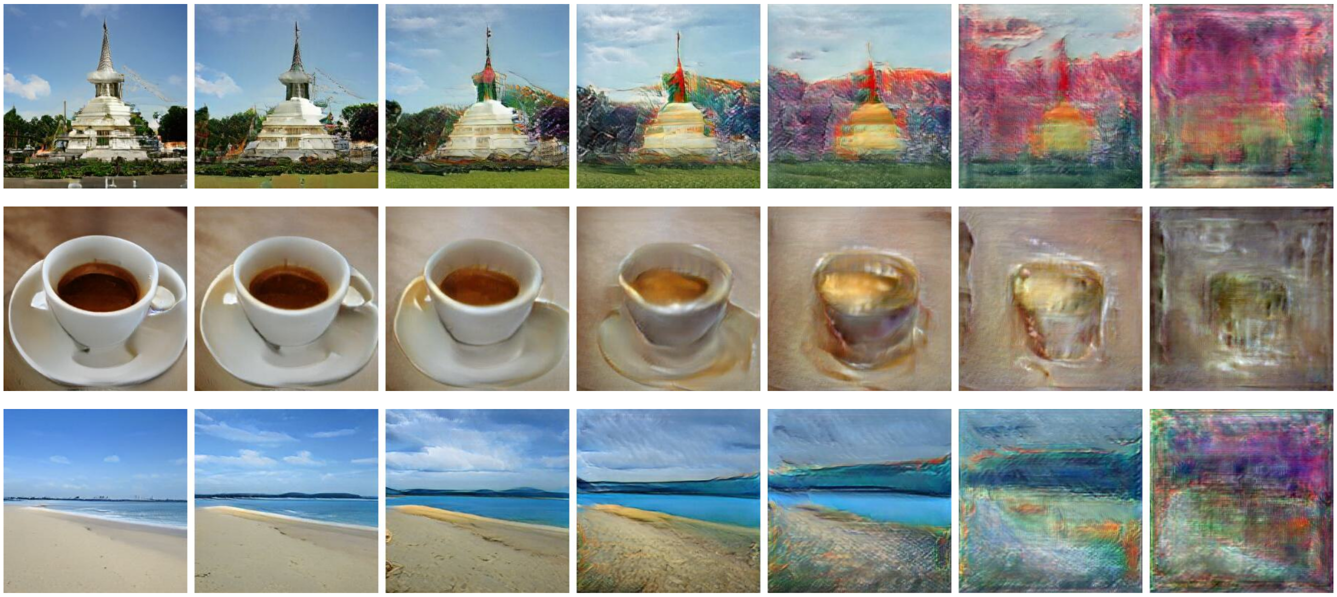

Figure S3. The variability of generated images as a function of the deformation parameter α (from left to right $\alpha = 0, 0.1, 0.2, 0.3, 0.4, 0.5, 0.6$). The value chosen throughout the paper is $\alpha = 0.35$.

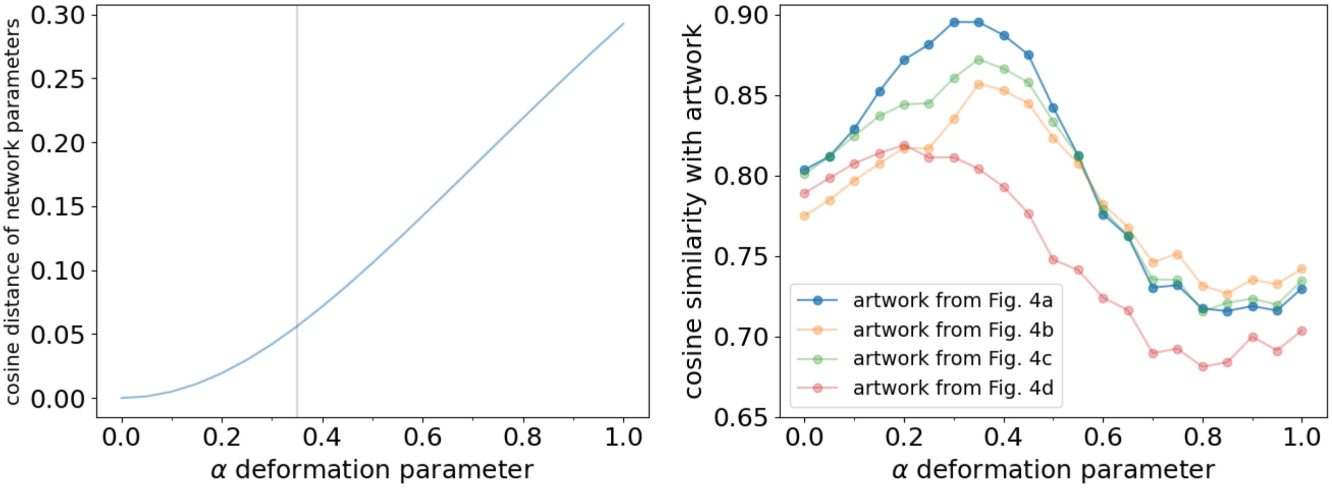

Figure S4. The relation between the deformation parameter α and the cosine distance in the 55-million dimensional space of `BigGAN` neural network parameters. The vertical line marks the value $\alpha = 0.35$ adopted in this paper (left panel). The perceptual similarity of the human made artworks from Fig. 4 with the counterparts of image Fig. 4a(left) generated for a range of the α deformation parameter (right panel). Some of the generated images used for this comparison are shown in the top row of Fig. S3.

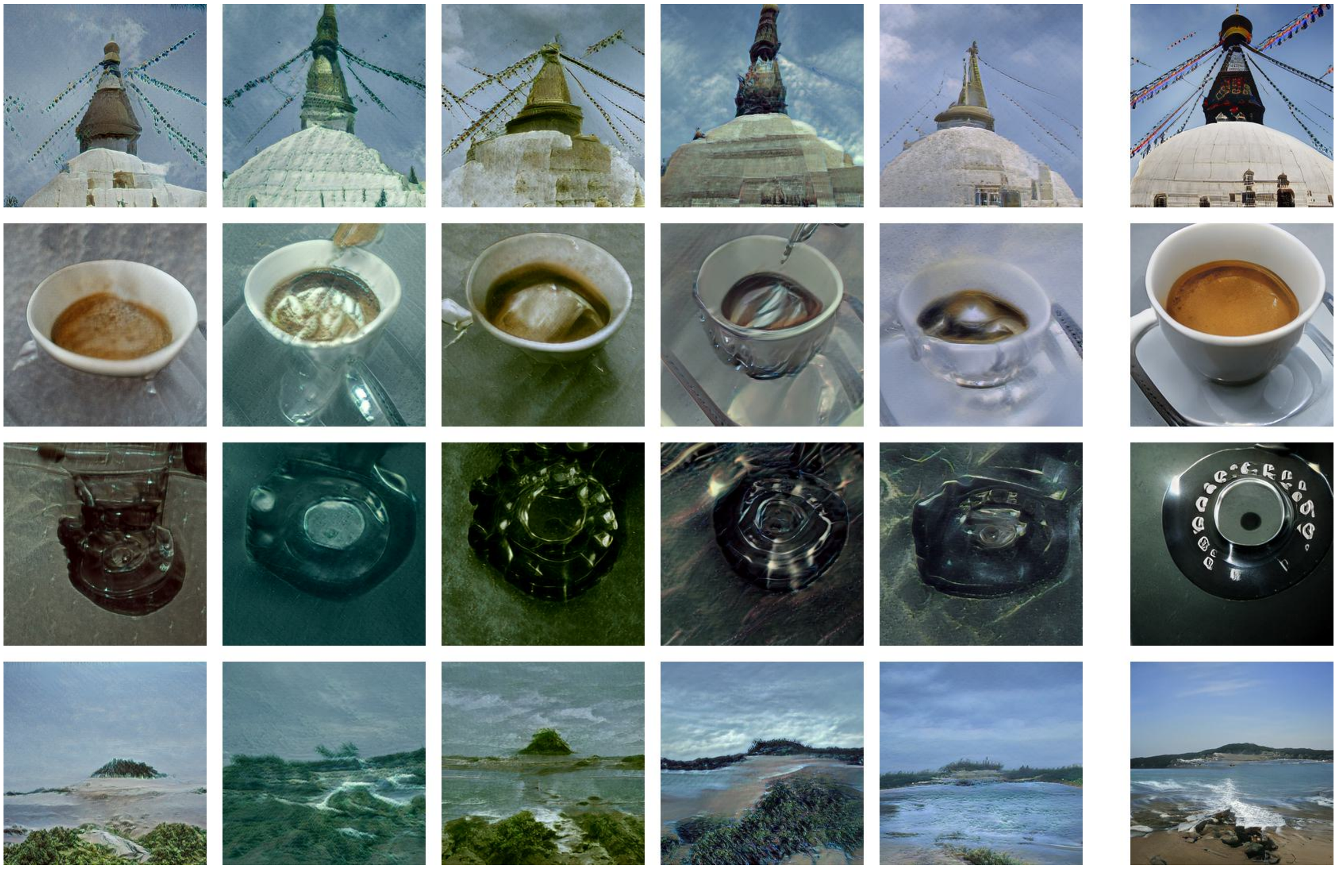

Figure S5. Images generated by networks of the `StyleGAN-XL` architecture with weights given by Eq. (3) in the main text. Each column corresponds to a particular random deformation (with seeds from the range 0-10). Images generated by the undeformed network are in the rightmost column.

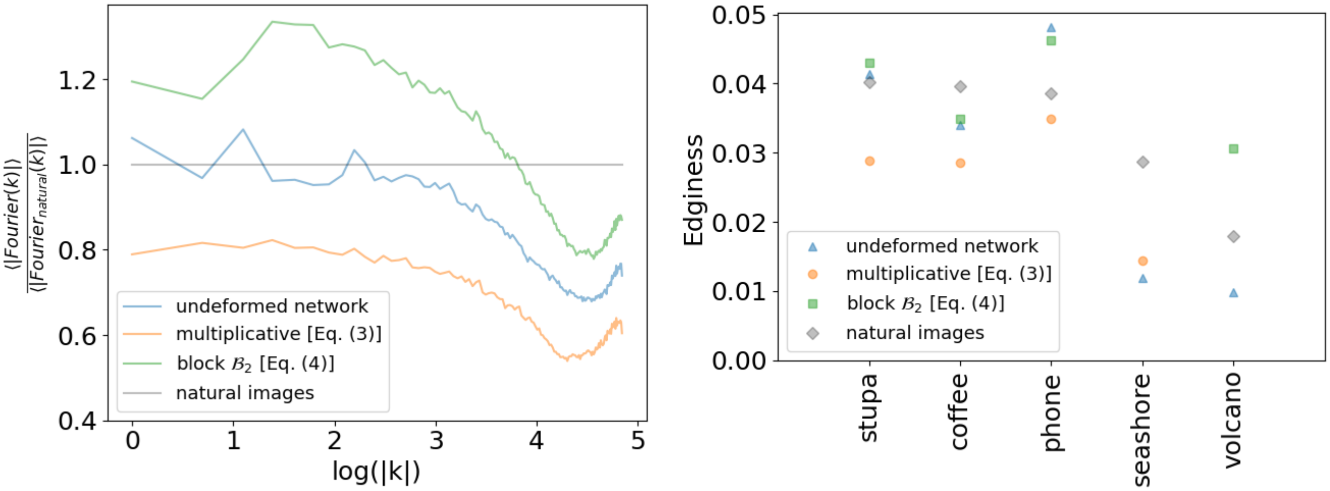

Figure S6. The ratio of the Fourier spectra of generated images to the Fourier spectra of corresponding natural images from the ImageNet dataset (left panel). A numerical characterization of the prominence of edges for natural and generated images of various types (right panel). We show results for the original undeformed network as well for the two types of deformations studied in the present paper.

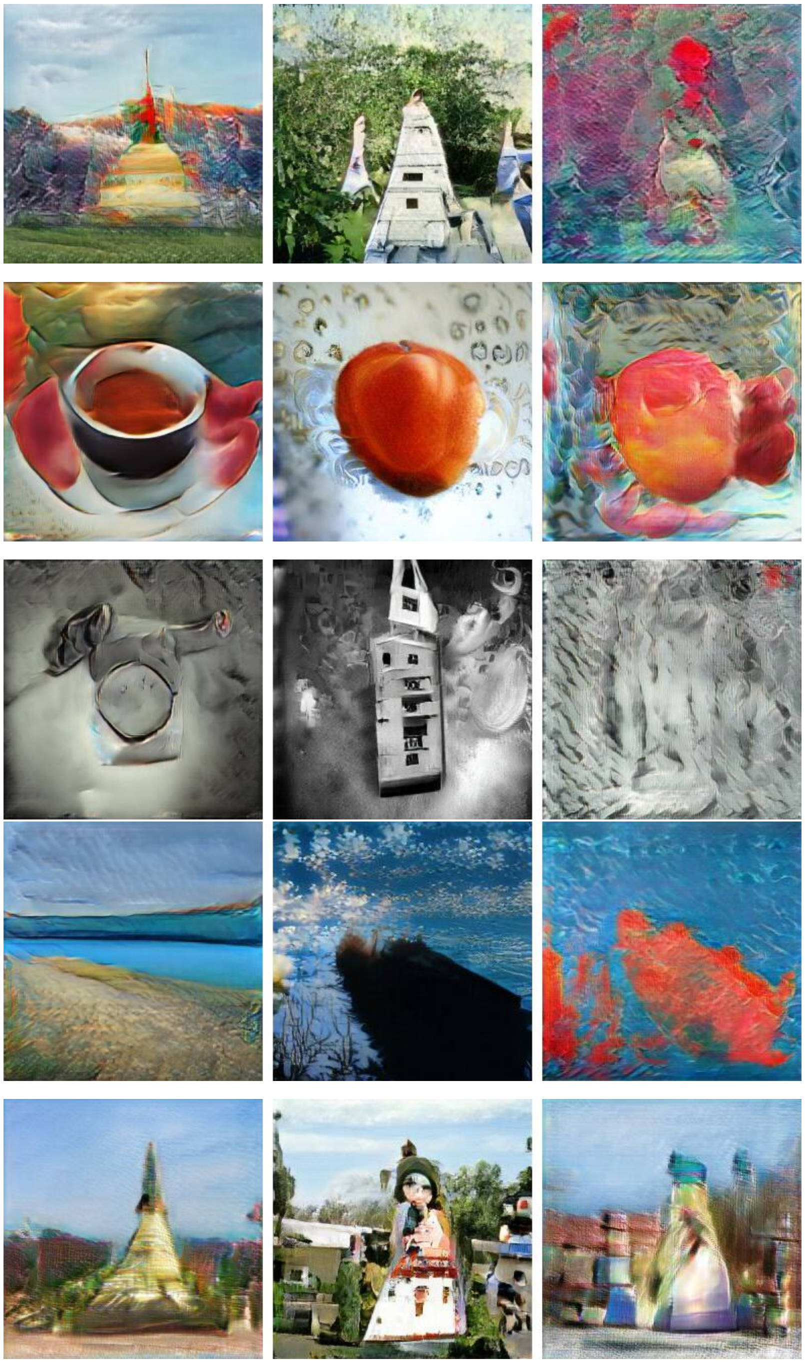

Figure S7. Nonuniform multiplicative deformations. Left column: standard (uniform) multiplicative deformation with $\alpha = 0.35$. Middle column: deformation with $\alpha = 2$ in block $\mathscr{B}_2$ and $\alpha = 0$ elsewhere. Right column: deformation with $\alpha = 2$ in block $\mathscr{B}_2$ and $\alpha = 0.35$ elsewhere. The random tensors entering the multiplicative deformations in each row are identical (they also coincide in the first four rows).
